# Identification of preoperative prediction factors of tumor subtypes for patients with solitary ground-glass opacity pulmonary nodules

**DOI:** 10.1186/s13019-018-0696-7

**Published:** 2018-01-17

**Authors:** Meishuang Li, Yanan Wang, Yulong Chen, Zhenfa Zhang

**Affiliations:** 1 Department of Lung Cancer, Tianjin Medical University Cancer Institute and Hospital, Huanhu West Road, Tianjin, 300060 China; 2 National Clinical Research Center for Cancer, Key Laboratory of Cancer Prevention and Therapy, Tianjin, 300060 China; 3 Tianjin’s Clinical Research Center for Cancer, Tianjin, 300060 China; 4 Tianjin Lung Cancer Center, Tianjin, 300060 China

**Keywords:** Solitory ground-glass opacity pulmonary nodules, ROC curve, Clinical features, Five-factor combination, Pathology

## Abstract

**Background:**

Recent wide spread use of low-dose helical computed tomography for the screening of lung cancer have led to an increase in the detection rate of very faint and smaller lesions known as ground-glass opacity nodules. The purpose of this study was to investigate the clinical factors of lung cancer patients with solitary ground-glass opacity pulmonary nodules on computed tomography.

**Methods:**

A total of 423 resected solitary ground-glass opacity nodules were retrospectively evaluated. We analyzed the clinical, imaging and pathological data and investigated the clinical differences in patient with adenocarcinoma in situ / minimally invasive adenocarcinoma and those with invasive adenocarcinoma.

**Results:**

Three hundred and ninety-three adenocarcinomas (92.9%) and 30 benign nodules were diagnosed. Age, the history of family cancer, serum carcinoembryonic antigen level, tumor size, ground-glass opacity types, and bubble-like sign in chest CT differed significantly between adenocarcinoma in situ / minimally invasive adenocarcinoma and invasive adenocarcinoma (*p*:0.008, 0.046, 0.000, 0.000, 0.000 and 0.001). Receiver operating characteristic curves and univariate analysis revealed that patients with more than 58.5 years, a serum carcinoembryonic antigen level > 1.970 μg/L, a tumor size> 13.50 mm, mixed ground-glass opacity nodules and a bubble-like sign were more likely to be diagnosed as invasive adenocarcinoma. The combination of five factors above had an area under the curve of 0.91, with a sensitivity of 82% and a specificity of 87%.

**Conclusion:**

The five-factor combination helps us to distinguish adenocarcinoma in situ / minimally invasive adenocarcinoma from invasive adenocarcinoma and to perform appropriate surgery for solitory ground-glass opacity nodules.

## Background

Recent widespread use of low-dose helical computed tomography (CT) for the screening of lung cancer have led to an increase in the detection rate of very faint and smaller lesions known as ground-glass opacity (GGO) nodules. GGO is a nonspecific finding that may be caused by various disorders, including inflammatory disease, hyperemia, focal fibrosis and neoplastic disease. The new interdisciplinary IASLC/ATS/ERS classification of lung adenocarcinoma has achieved a considerable impact since its publication in the year 2011. It puts forward that the preinvasive lesions atypical adenomatous hyperplasia (AAH) and adenocarcinoma in situ (AIS) together with minimally invasive adenocarcinoma (MIA) have an excellent prognosis after complete resection with 100% survival or approaches 100% survival. Several recent studies have demonstrated comparable recurrence and survival rates for lobectomy and sublobar resection, even in good-risk patients with small stage I lung cancer [1–4]. A GGO appearance on chest CT has been reported to be associated with a favorable histology such as non-or minimally invasive adenocarcinoma in lung cancer [5]. These GGO lesions are also likely to be amenable to sublobar resection. Serum carcinoembryonic antigen (CEA) is a useful circulating biomarker and now well-known and validated serum biomarker for lung cancer. Maeda et al. [6] reported that CEA level was an important clinical predictor of tumor invasiveness in patients with clinical stage IA non-small cell lung cancer (NSCLC).

Although some studies have identified some clinical and imaging factors and used the combination of the selected parameters for the preoperative prediction of tumor subtypes in patients with T1 lung cancer, there is no report about solitary GGO nodules on chest CT. Therefore, the purpose of our study was to investigate parameters that preoperatively predicted histological subtypes in patients with solitary GGO nodules on chest CT.

## Methods

### Study population

This study was approved by the institutional review board of Tianjin Medical University Cancer Institute and Hospital, Tianjin, China. Between January 2013 and December 2016, 6317 patients with pulmonary nodules underwent surgical resection with curative intent at Cancer Institute and Hospital of Tianjin Medical University. Of these patients, 423 were selected according to inclusion criteria and exclusion criteria. Our inclusion criteria were as follows: (1) patients with solitary GGO nodules on chest CT scan, (2) patients who had their lesions surgically removed and had postoperative pathological diagnosis, (3) R0 resection. Our exclusion criteria included: (1) patients who had no CT scan, (2) pulmonary multiple GGO nodules, mixed GGO with consolidation to the maximum tumor diameter greater than 0.75 or pure solid tumors, (3) patients with history of lung cancer or who had a malignancy elsewhere. All patients underwent lobectomy or limited resection (segmentectomy or wedge resection) with hilar and mediastinal lymphadenectomy or lymph node sampling. There were no objective criteria for limited resection, and the indications depended on each surgeon’s preference. A patient with a GGO > 5-8 mm may be subjected to surgical treatment and with a GGO less than 20 mm may be given the limited resection, including segmentectomy and wedge resection.

### Clinical and pathological characteristics

For each patient, age, gender, smoking status, a family history of cancer, location of tumor, the serum tumor markers: carcinoembryonic antigen (CEA) and carbohydrate antigen 19–9 (CA19–9) and histological subtypes were extracted from patient medical records. Classification of lung adenocarcinoma was assessed by two pathologists in accordance with the new interdisciplinary IASLC/ATS/ERS classification of lung adenocarcinoma. We classified all patients into two groups: AIS /MIA group and invasive adenocarcinoma (IA) group.

### CT imaging

Chest CT scans were performed before surgery by using one of three multi-detector CT systems: Somatom Sensation 64 (Siemens Medical Solutions, Forchheim, Germany), Light speed 16, and Discovery CT750 HD (GE Healthcare, Milwaukee, WI, USA) scanner. Scanning parameters were as follows: 120kVp with tube current adjusted automatically, 1.5 mm reconstructionthickness with 1.5 mm reconstruction interval for 64-detector scanner; and 120kVp, 150–200 mA, 1.25 mm reconstruction thickness with 1.25 mm reconstruction interval for the other two scanners. GGO was defined as a hazy increase in lung attenuation without obscuring the underlying vascular marking. Two observers who were unaware of pathologic staging viewed each CT scan of 423 solitary GGO nodules and assessed nodules morphology blindly. Morphology included density, size, air bronchogram, bubble-like sign, spicule sign, pleural tag or lobular. Based on the density via CT, GGO nodules were classified into two groups: pure GGO (pGGO) (a tumor without solid component), and mixed GGO (mGGO) (a tumor with both GGO and solid components).

### Blood specimen collection and measurement

About 3 ml of peripheral blood was collected from each case in coagulated tube. The serum was separated by centrifuging at 3000×g for 5 min, and then transferred to a new Eppendorf tube, and stored at − 80 °C for further analysis. Tumor biomarkers, including CEA, CA19–9 were measured using electrochemiluminescence immunoassays according to standard procedure of Roche Company’s kit and Roche E170 automatic immunity analyzer. The cut-off points for each tumor biomarker determined by the manufacturer, were as follows: CEA, 5 μg/l; CA19–9, 39 U/ml.

### Statistical analysis

Statistical analysis performed by using SPSS 24.0 software. The T test, Mann-Whitney U test, χ^2^ test or Fisher’s exact test were used to test for difference in clinical factors and imaging characters between different pathology groups, asappropriate. T test was used for categorical variables fitted normal distribution and expressed by x ± s. Mann-Whitney U test was used for categorical variablesfitted non-normal distribution and expressed by M (P25, P75). χ^2^ test or Fisher’s exact test was used for categorical variables. Receiver operating characteristics (ROC) curves were generated to evaluate the predictive potential of identified clinical and imaging signatures for IA, then combined all identified factors to predict histological types by adding all five factors to a bivariable-adjusted logistic regression model. Optimal cut-off values were calculated by ROC cures. Univariate logistic regression and binary logistic regression analysis were also performed to assess the diagnostic accuracy by using cut-off values. All statistical tests were two-sided, and a *p* value ≤0.05 was considered statistically significant.

## Results

### Patients demographics

There were total 423 patients with solitory GGO nodules. Among them, 393 had adenocarcinoma and 30 had benign nodules. The clinicopathologic characteristics of 393 (92.9%) patients with adenocarcinoma were summarized in Table 1. There were 117 male and 276 female patients (median age, 57 years; range: 27–78 years). Two hundred and ninety-six of 393 (75.3%) patients were never active smokers. Sixty-six patients had a family history of other cancers (such as liver cancer, gastric cancer, colorectal cancer and bone cancer) and 50 (43.1%) patients had a family history of lung cancer. Of 393 patients, preoperative serum CEA and CA19–9 were tested in 379 patients. The number of patients with elevated CEA and CA19–9 were 25 (6.6%) and 81 (21.4%), respectively. GGO nodules were often found in the superior lobe of right lung (*n* = 163), followed by the superior lobe of left lung (*n* = 98). The histological types according to IASLC/ATS/ERS classification were as follows: 269 (68.4%) patients with IA, 115 (29.3%) patients with MIA, 9 (2.3%) patients with AIS. Lobectomy, segmentectomy and wedge resection were performed in 349 (88.8%), 21 (5.3%) and 23 (5.9%) patients, respectively.

**Table 1:** Characteristic of the patients with GGO

Variable	Number (%)
Gender	
Male	117 (29.8)
Female	276 (70.2)
Age	
≤ 60	233 (59.3)
> 60	160 (40.7)
Smoking	
Current/ever	97 (24.7)
Never	296 (75.3)
Family history of cancer	
Yes	116 (29.5)
No	277 (70.5)
Tumor marker	
CEA(+)	25 (6.6)
CA19–9(+)	4 (1.1)
Surgical method	
Lobectomy	349 (88.8)
Segmentectomy	21 (5.3)
Wedge resection	23 (5.9)
Pathological type	
AIS/MIA	9/115 (31.6)
IA	269 (68.4)
Pathological stage	
0	9 (2.3)
IA	370 (94.1)
IB	14 (3.6)
Lymphatic metastasis	0

Abbreviation: *CEA* carcinoembryonic antigen, *CA19–9* carbohydrate antigen 19–9, *AIS* adenocarcinoma in situ, *MIA* minimally invasive adenocarcinoma, *IA* invasive pulmonary adenocarcinoma

### Clinical and imaging factors that predict histological subtypes

**Fig. 1:**
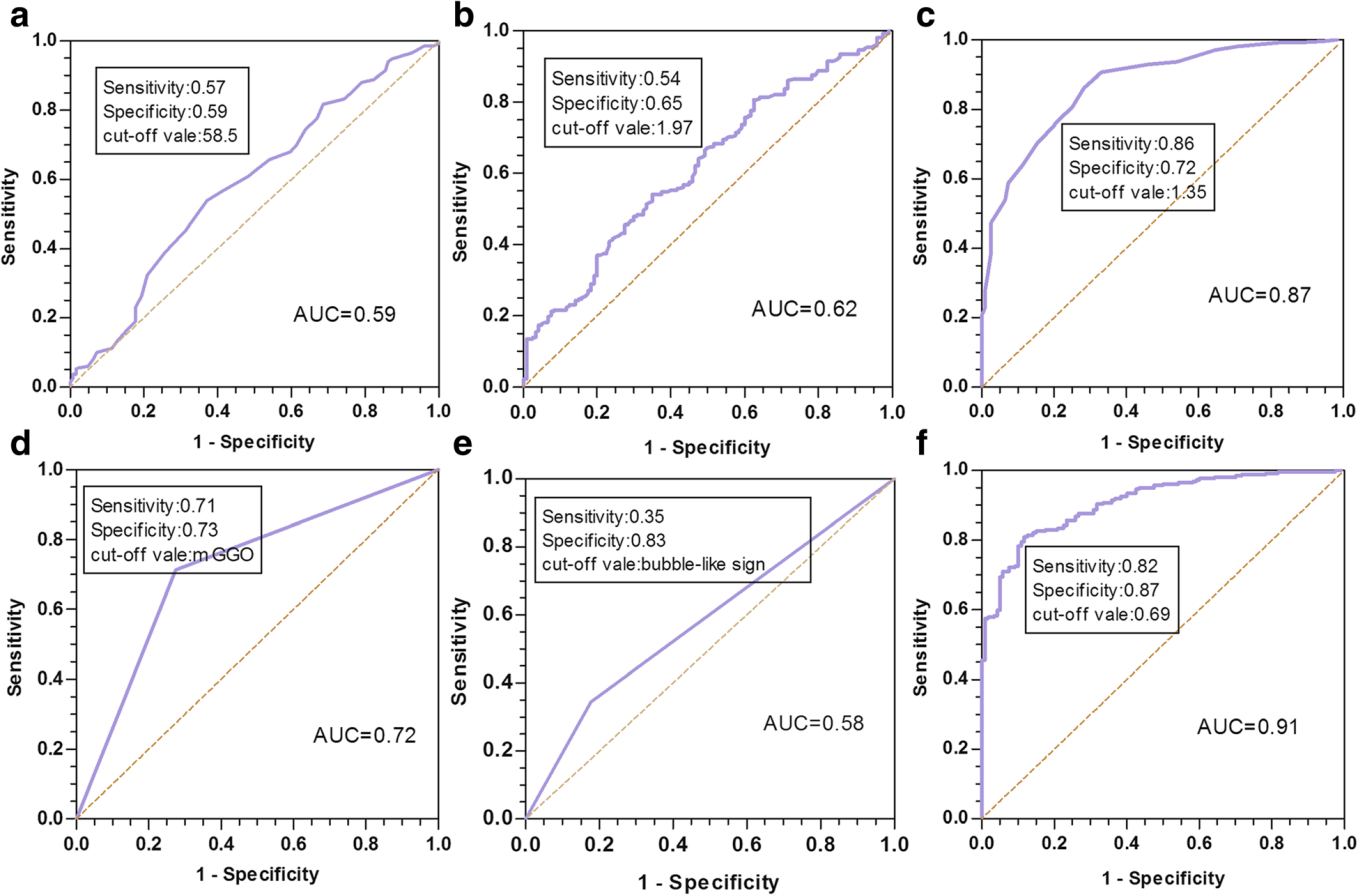
Receiver operating characteristic (ROC) curves assessing the accuracy of clinical and imaging factors in predicting invasive adenocarcinoma (IA) versus adenocarcinoma in situ (AIS) or minimally invasive adenocarcinoma (MIA). The *p* value for age (**a**), serum carcinoembryonic antigen (CEA) level (**b**), tumor size (**c**), ground-glass opacity type (**d**), Bubble-like sign (**e**) and the combination of these five factors (**f**) were 0.005816, 0.00020, < 0.0001, < 0.0001, 0.001and < 0.0001, respectively

Age, family history of cancer, Serum CEA level, GGO size, type of GGO and bubble-like sign differed significantly between patients with AIS/MIA and those with IA (*p*: 0.008, 0.046, 0.000, 0.000, 0.000, and 0.001). (Table 2). By the bivariate logistic analysis, tumor size, mixed GGO and bubble-like sign were independent predictors of IA (*p*:0.000, 0.000 and 0.021, respectively). ROC curves were generated to assess the IA prediction accuracy of the six factors identified in univariate analysis. It showed that a family history of cancer had a poor accuracy (*p* = 0.1148). Therefore, this parameter was not brought into muti-factor combination. Figure 1 shows the true-positive ratios (sensitivity) and false-positive ratios (1 minus specificity) for age, CEA, tumor size, GGO type and bubble-like sign. The areas under curves (AUCs) for age, serum CEA level, tumor size, GGO type and bubble-like sign were 0.59, 0.62, 0.87, 0.72, and 0.58 (*p*: 0.0058, 0.0002, < 0.0001, < 0.0001, and 0.001, respectively), with sensitivities of 57%, 54%, 86%, 71%, 35% and specificities of 59%, 65%, 72%, 73%, 83%. We also assessed the IA prediction accuracy of these five factors in combination via bivariate logistic regression analysis. The AUC of the five-factor combination was 0.91, with a sensitivity of 82% and a specificity of 87% (*p* < 0.0001). (Table 3). To further distinguish the IA from AIS/MIA, we performed univariate and multivariate analyses using the optimal cut-off values calculated from the ROC curves (Table 4). According to the univariate analysis, patients were more likely to be diagnosed with IA if they had these factors: more than 58.5 years, a serum CEA level > 1.970 μg/L, a tumor size > 13.50 mm, mGGO and bubble-like sign (*p*: 0.005, 0.001, 0.000, 0.000, and 0.001, respectively). According to the bivariate analyses, the combination of these five factors was an independent diagnostic factor for IA (*p*:0.000).

**Table 2:** Correlation between histological subtypes and clinical and CT imaging characteristics

Variable	AIS/MIA(124)	IA(269)	*p* value
Gender			0.984
Male	37	80	
Female	87	189	
Median age	55.85 ± 9.42	58.49 ± 9.00	0.008*
Smoking			0.246
Current/ever	98	198	
Never	26	71	
History of family cancer			0.046*
Yes	45	71	
No	79	198	
CEA	1.5 (1.0,2.4)	2.1 (1.3,3.1)	0.000*
CA19–9	11.0 (8.4,17.4)	9.4 (6.5,15.3)	0.081
Location of tumor			0.084
RUL	52	111	
RML	8	12	
RLL	15	50	
LUL	39	59	
LLL	10	37	
GGO size	1.2 (0.8,1.6)	2.0 (1.6,2.5)	0.000*
GGO type			0.000*
pGGO	90	77	
mGGO	34	192	
Air bronchogram	18	71	0.074
Bubble-like sign	22	92	0.001*
Spicule sign	32	79	0.140
Pleural tag	31	71	0.163
Pathological stage			0.000*
0/IA	124	255	
IB	0	14	
Lymphatic metastasis	–	–	–

^*^Statistically significant *p* value, *CEA* carcinoembryonic antigen, *CA19–9* carbohydrate antigen 19–9, *pGGO* pure ground-glass opacity nodule, *mGGN* mixed ground-glass opacity nodule, *RUL* superior lobe of right lung, *RML* middle lobe of right lung, *RLL* inferior lobe of right lung, *LUL* superior lobe of left lung, *LLL* inferior lobe of left lung

**Table 3:** Results of ROC curves assessing the accuracy of clinical and imaging factors in predicting IA versus AIS / MIA

variable	Area	95% CI	Sensitivity	Specificity	Cut-off vale	*p* value
Tumor size	0.87	(0.83,0.91)	0.86	0.72	1.35	<0.0001
Age	0.59	(0.53,0.65)	0.57	0.59	58.50	0.005816
History of family cancer	0.55	(0.49,0.61)	0.74	0.36	0.66	0.1148
CEA	0.62	(0.56,0.68)	0.54	0.65	1.97	0.00020
GGO type	0.72	(0.66,0.77)	0.71	0.73	0.66	<0.0001
Bubble-like sign	0.58	(0.53,0.64)	0.35	0.83	0.63	0.00100
Five-factor combination	0.91	(0.88,0.94)	0.82	0.87	0.69	<0.0001

Five factors including tumor size, age, serum CEA level, GGO type and Bubble-like sign

**Table 4:** Univariate and bivariate analysis to predict pathological subtype using optimal cut-off values

Variable			Univariate			Bitivariate	
	Cut-off vale	OR	95%CI	*p* value	OR	95%CI	*p* value
GGO size	>13.50	15.944	(9.453,26.894)	0.000	2.413	(0.845,6.812)	0.093
Age	>58.50	1.860	(1.208,2.862)	0.005	1.650	(0.872,3.136)	0.106
CEA	>1.97	2.185	(1.396,3.419)	0.001	1.217	(0.638,2.305)	0.536
GGO type	mGGO	2.569	(2.026,3.258)	0.000	1.446	(0.917,2.283)	0.112
Imaging feature	Bubble-like sign	1.936	(1.258,4.263)	0.001	1.213	(0.645,2.134)	0.127
Five-factor combination	0.69	313.679	(107.5868.8)	0.000	58.238	(7.536,440.632)	0.000*

* Statistically significant *p* value

## Discussion

Limited resection including segmentectomy and wedge resection has been recently advocated for patients with AIS or MIA due to its preservation of lung function. But it is not suitable for patients with IA. Thus, it would be helpful to use preoperative factors to predict the histological type of GGO because many GGO nodules were AIS or MIA. Due to the low cellularity in GGO lesions, the diagnostic yield of percutaneous needle biopsy for GGO lesions was reported to be significantly lower than that of solid lesions [7]. Therefore, circulating tumor marker levels and CT imaging are attractive alternatives.

Numerous studies proved that the CT findings were useful for evaluating the histological nature of the tumors and correlated with the IASLC/ATS/ERS classification [8–10]. Our results showed that benign lesions only accounted for 7.1%, the reason may be the selection of candidate patients for surgery. Because we usually resected GGNs which were more likely to be malignant, such as larger tumor diameter (more than 8 mm), mGGO with solid contents, changing of diameter or contents of GGNs during follow-up. From a survey of 492 lung cancers of all pathological types and stages, Seki et al. [11] reported that GGO was found only in adenocarcinoma. In line with these findings, our results showed that all patients with pulmonary GGO nodules are confirmed to be lung adenocarcinoma. Generally, the larger the nodules are, the more likely they will be IA. Our study revealed that tumor size was an independent predictive factor for IA. We distinguished IA from AIS/ MIA when an optimal cut-off value of 13.50 mm was used. Lee et al. [12] reported that optimal cut-off values of 10 mm and 14 mm for distinguishing preinvasive lesions from invasive pulmonary lesions in cases of pGGO and mGGO, respectively. However, a study recommended that 11 mm was the tumor size cut-off value for differentiating IA from AIS and MIA in patients with T1 lung adenocarcinoma.

A study reported that lesions with GGO appearance were more likely to be “early” adenocarcinoma such as BAC, AIS, or MIA, whereas more advanced adenocarcinomas include a larger solid component within the GGO region [13, 14]. Our study revealed that a solid component was associated with IA.. In our study, the presence of mGGO nodule predicted IA with an AUC of 0.72. Although our study showed that CT findings were useful diagnostic factors of pathological types, other factors should be identified to improve sensitivity and specificity.

Serum biomarker as a diagnostic tool with less invasive and rapid detection was widely used for malignant tumor. Serum CEA is a useful circulating biomarker and now a well-known and validated serum biomarker for lung adenocarcinoma. However, the optimal cut-off value for serum CEA level varies in the literature [15]. Using the optimal cut-off value identified in our study (> 1.970 μg/L,), We found CEA was associated IA in patients with GGOs.

A significant difference was also noted in age, which could be explained by the hypothesis of sequential development of small AAH to adenocarcinoma. However, it was sometimes unreasonable to accurately predict the pathological types using only a single factor in patients with GGO nodules. To possibly improve accuracy, we combined the five factors (age, serum CEA level, GGO type, tumor size, and bubble-like sign) that distinguished AIS/MIA from IA preoperatively. Therefore, if patients have the following parameters: age ≤ 58.5 years, serum CEA level ≤ 1.970 μg/L, tumor size ≤13.5 mm, pGGO, and without a bubble-like sign in chest CT scan, limited resection was suggested.

There are limitations of this study. First, this was a single institution retrospective analysis and the number of patients was small. Second, all patients in our study were resected within 4 years. We didn’t make the survival analysis because patients with GGOs had excellent prognoses. Finally, variations in nodule measurement and characterization of lesions might be possible due to different observers.

## Conclusion

The results revealed that the persistent presence of a solitary GGO nodule may be lung adenocarcinoma. Our results successfully validated potential usefulness of serum CEA level, tumor size and GGO type and bubble-like sign in predicting pathological types in patients with solitary GGO pulmonary nodules. The five-factor combination helps us to distinguish AIS/MIA from IA in patients with GGO and to perform an appropriate surgical resection.

## Abbreviations

AAH: Atypical adenomatous hyperplasia
AIS: Adenocarcinoma in situ
AUCs: Area under curves
CA-19-9: Carbohydrate antigen 19–9
CEA: Carcinoembryonic antigen
GGO: Ground-glass opacity
IA: Invasive adenocarcinoma
mGGO: Mixed GGO
MIA: Minimally invasive adenocarcinoma
NSCLC: Non-small cell lung cancer
pGGO: Pure GGO
ROC: Receiver operating characteristics
